# Dual‐Band Modulation of Emissivity and Solar Transmittance Through Chemical Composition‐Tuned Asymmetric Layer‐by‐Layer Assembly

**DOI:** 10.1002/advs.74461

**Published:** 2026-02-20

**Authors:** Hebing Hu, Zhengui Zhou, Guanya Wang, Xiaofei Li, Yun Meng, Tao Xu, Xiao'e Jia, Hao Yu, Yuhan Wang, Jiarui Wang, Yi Long

**Affiliations:** ^1^ International Institute for Materials Innovation Nanchang University Nanchang China; ^2^ Department of Electronic Engineering The Chinese University of Hong Kong Hong Kong SAR China; ^3^ School of Physics and Electronics Shandong Normal University Jinan China; ^4^ School of Materials Science and Engineering Nanyang Technological University Singapore Singapore

**Keywords:** asymmetric layer‐by‐layer assembly, energy saving, infrared‐transmitting spacers, spectral selectivity, superstructures

## Abstract

Buildings contribute approximately 40% of global energy consumption, with windows being among the least energy‐efficient and most complex components of building envelopes. Recent advancements in vanadium dioxide (VO_2_)‐based Fabry‐Pérot (F‐P) resonators, which integrate both solar modulation and radiative cooling (RC) regulation into a single, energy‐efficient window, present a significant opportunity for year‐round global energy conservation. However, the effectiveness of this innovation is often limited by inadequate modulation capabilities and low luminous transmittance (*T*_lum_) resulting from spatial disorder at the nanoscale. In this study, we introduce an unconventional asymmetric Layer‐by‐Layer (LbL) assembly technique that spatially leverages wavelength‐specific functional components to enhance spectral selectivity. This approach achieves a 52.1% improvement in *T*_lum_ and an 8.6% increase in solar modulation (Δ*T*_sol_) compared to current state‐of‐the‐art technologies, while maintaining a comparable mid‐infrared (MIR) emissivity modulation (Δ*ε*_MIR_) of 0.4. These findings validate the efficacy of the LbL process in fabricating spectrally selective smart devices and underscore its significant potential beyond just energy‐efficient smart windows.

## Graphical abstract

An unconventional asymmetric Layer‐by‐Layer (LbL) assembly technique was developed to fabricate a Fabry‐Pérot (F‐P) resonator, which enhances luminous transmittance (*T*_lum_) and enables dual‐band modulation in both the near‐infrared (NIR) and mid‐infrared (MIR) regions. This superstructure induces light interference effects, thereby improving both *T*_lum_ and solar modulation (Δ*T*_sol_). It demonstrates impressive emissivity regulation (Δ*ε*_MIR_) of 0.4 and excellent energy‐saving performance.

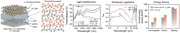

## 1 Introduction

Buildings account for a significant portion of global energy consumption, with a substantial fraction attributed to heating, ventilation, and air conditioning (HVAC) systems [1]. Windows, as integral components of building envelopes, play a crucial role in regulating indoor thermal comfort; however, they often lead to excessive energy loss due to poor thermal insulation and uncontrolled solar heat gain [2, 3, 4, 5, 6, 7]. To address this challenge, smart windows with dynamic optical properties have emerged as a promising solution for energy‐efficient buildings. Conventional smart windows primarily focus on modulating visible light or near‐IR (NIR) solar radiation to control solar heat gain [5, 6, 7, 8, 9, 10, 11, 12, 13]. Additionally, passive RC windows with high *ε*_MIR_ offer an alternative approach for energy conservation by rejecting heat in the form of MIR waves to the cold outer space [14, 15, 16, 17, 18, 19, 20, 21]. The recent development of energy‐saving windows that can modulate both solar transmittance (*T*_sol_) and *ε*_MIR_ is highly desirable for reducing year‐round energy consumption [14].

**FIGURE 1:**
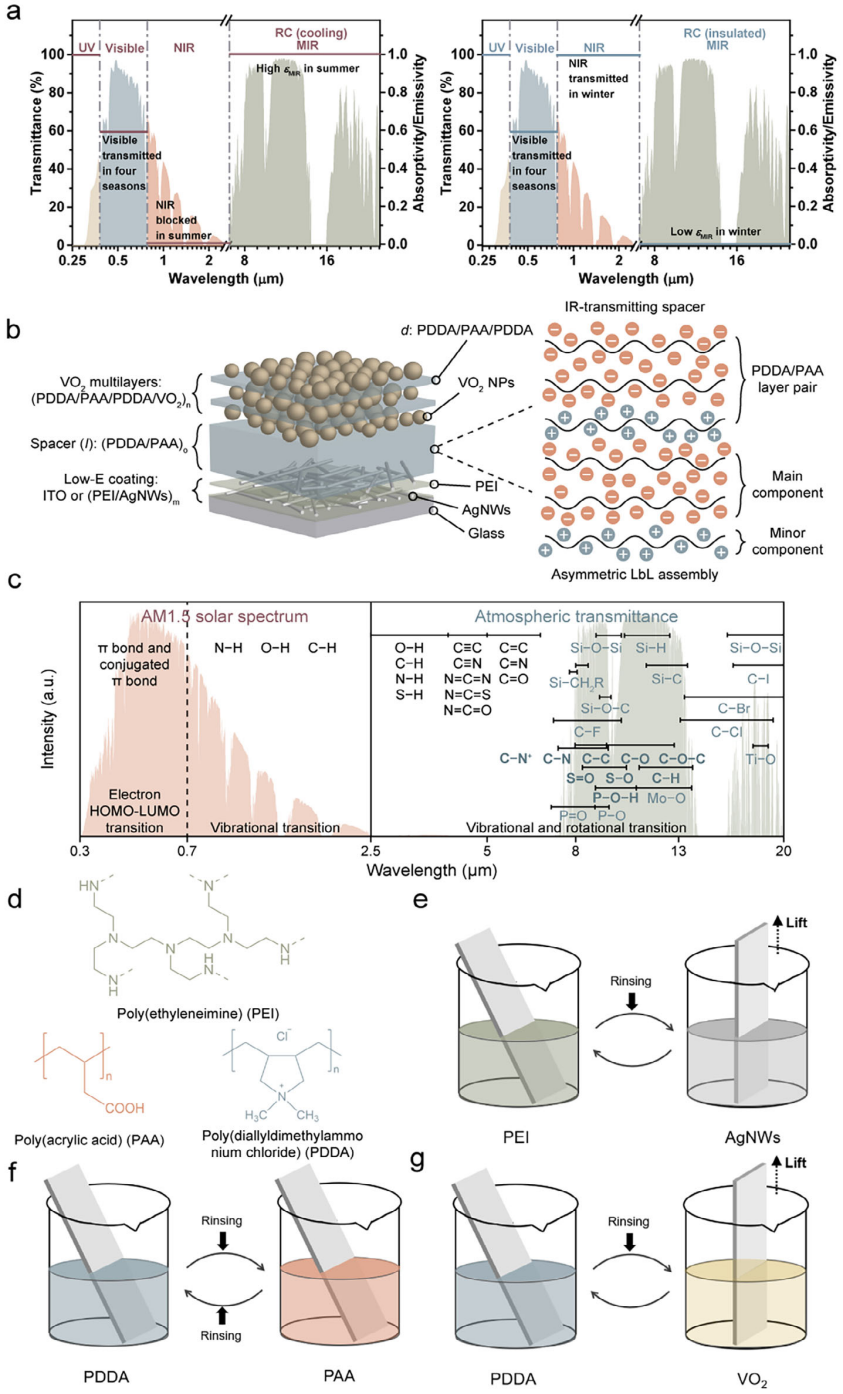
(a) Concept of the ideal RC window. The red and blue lines represent the spectral behavior of an ideal RC window in summer (left) and winter (right). Visible light is transmitted throughout all seasons, while NIR light is blocked in summer and transmitted in winter. The window is designed to exhibit high *ε*_MIR_ in summer and low *ε*_MIR_ in winter. (b) Schematic representation of the LbL‐assembled RC window fabricated using an asymmetric LbL assembly approach. This window features a F‐P cavity structure consisting of a low *ε*_MIR_ coating, an IR‐transmitting spacer, and multilayers of VO_2_. By diluting the concentration of PDDA during the LbL assembly process, the proportion of the PAA component increases while the proportion of the PDDA component decreases within the (PDDA/PAA)_o_ spacer. (c) Spectra of chemical bonds or functional groups within the range of 0.3 to 20 µm. The normalized AM 1.5 Global (AM1.5G) solar spectrum (indicated by the red shaded area) and the atmospheric transmission spectrum (indicated by the green shaded area) are provided as references. (d) Molecular structures of the polyelectrolytes used for LbL assembly. (e) Schematic representation of the dip‐coating process for the low *ε*_MIR_ coatings of (PEI/AgNWs)_m_. (f) Schematic representation of the dip‐coating process for the (PDDA/PAA)_o_ spacers. (g) Schematic representation of the dip‐coating process for the (PDDA/VO_2_)_n_ structures.

However, achieving such complex spectral selectivity presents significant challenges in structural design and process control. Existing strategies are often constrained by intricate structural configurations (e.g., interwoven architectures [22]) or by high‐cost fabrication processes, such as photolithography [23] and magnetron sputtering [24, 25]. Furthermore, optimizing key optical parameters, including high *T*_lum_, Δ*T*_sol_, and Δ*ε*_MIR_ (Figure 1a), is complicated by the absence of precise fabrication methods capable of controlling both the quantity and spatial arrangement of wavelength‐specific functional components within the structure.

LbL assembly, a well‐established and widely used technique [26, 27, 28, 29, 30, 31, 32], offers distinct advantages in fabricating large‐area and homogeneous coatings with unique optical [33, 34, 35], mechanical [36, 37, 38], biomedical [39], electrical [40, 41], and magnetic [42] properties. A diverse range of components, including polymers [43, 44, 45, 46, 47, 48, 49], nanoparticles (NPs) [35, 50], nanowires (NWs) [34, 40, 51], nanosheets [52], nanofibrils [53], and proteins [39], can be routinely combined into an LbL film with controlled multilayer sequences and spatial arrangements. In this work, we aim to enhance *T*_lum_ and achieve dual‐band modulation in both the NIR and MIR regions by preparing a superstructure that incorporates F‐P resonance properties through LbL assembly (Figure 1b). In this superstructure, the functional groups within the spacer play a crucial role. An ideal spacer must fulfill two key functionalities: 1. To enhance the performance of the F‐P resonator, the spacer should exhibit high MIR transmittance while minimizing the incorporation of bonds such as C─N, C─N^+^, C─O─C, C─C, S═O, S─O, C─O, Mo─O and P═O, as these bonds have significant vibrational and rotational transitions that can lead to high absorption (Figure 1c) [18]; 2. The components should possess charged functional groups to facilitate the LbL assembly process.

Among the commonly used polyanions for LbL assembly, including poly(acrylic acid) (PAA), poly(sodium 4‐styrenesulfonate) (PSS), and carboxymethyl cellulose (CMC), PAA is the optimal choice for fabricating the F‐P cavity due to its low absorption in the MIR range of 8 ‐14 µm (1250 – 714 cm^−1^) (Figure 1d; Figure S1). In contrast, PSS exhibits strong absorption characteristics, attributed to the asymmetric and symmetric S═O stretching of the ─SO_3_^−^ group at 1200 – 1180 and 1060 ‐1040 cm^−1^, as well as S─O stretching near 1120 cm^−1^. CMC also demonstrates significant absorption due to intense C─O─C stretching in the range of 1050 – 1030 cm^−1^ (Figure S1). Among common polycations, poly(ethyleneimine) (PEI) shows a strong absorption peak from C─N stretching that overlaps with C─C skeletal vibrations at 1150 – 1100 cm^−1^. Poly(allylamine hydrochloride) (PAH) exhibits stretching vibrations of the C─N bond within the range of 1250 – 1000 cm^−1^. Poly(diallyldimethylammonium chloride) (PDDA) was selected as the polycation for LbL assembly because it features only a C─N^+^ stretching peak at 1100 – 950 cm^−1^ (Figure 1d; Figure S1).

In contrast to the conventional (symmetric) LbL assembly, which typically results in a similar amount of oppositely charged components, a unique asymmetric LbL assembly approach was developed. For the first time, this method was employed to fabricate the F‐P resonator (Figure 1b,e,f,g; Figure S2) and to minimize the overall MIR absorption of the spacer. This resulted in the formation of superstructures defined as either glass/(PEI/AgNWs)_m_/(PDDA/PAA)_o_/(PDDA/PAA/PDDA/VO_2_)_n_ (*m*, *o* or *n* = 1, 2, 3…) or glass/ITO/(PDDA/PAA)_o_/(PDDA/PAA/PDDA/VO_2_)_n_ (*o* or *n* = 1, 2, 3…). The underlying layers of silver nanowires (AgNWs) or indium tin oxide (ITO) serve as the low *ε*_MIR_ coatings, while the top multilayers of VO_2_ enable effective modulation of solar transmittance. Additionally, a tunable (PDDA/PAA)_o_ spacer between these layers facilitates *ε*_MIR_ regulation. Compared to our previously fabricated F‐P resonator using spin‐coating [14], the current LbL‐assembled superstructures can further optimize three key optical parameters, as LbL assembly allows for precise control over the quantity and spatial arrangement at the nanoscale. These LbL‐assembled superstructures demonstrated superior optical properties (*T*_lum_: 41%; Δ*T*_sol_: 10.1%; Δ*ε*_MIR_: 0.4) compared to previously reported windows, such as ITO/polymer/VO_2_ [14], polymer/VO_2_ composites [54], ITO/TiO_2_/VO_2_ [24], ITO/ZnSe/VO_2_ [55], and Al/SiO_2_/VO_2_/Al_2_O_3_ [25]. Both experimental and theoretical results confirm that the IR‐transmitting properties of the spacer layer play a critical role in RC regulation. Additionally, these findings underscore the importance of maintaining a well‐controlled interlayer spacer (*d*) between the VO_2_ layers, as this induces light interference effects that enhance both *T*_lum_ and Δ*T*_sol_. The energy‐saving performance of this superstructure across various climate zones highlights its potential as an effective solution for energy‐efficient windows.

## 2 Results and Discussions

### 2.1 Characterization of F‐P Resonator

**FIGURE 2:**
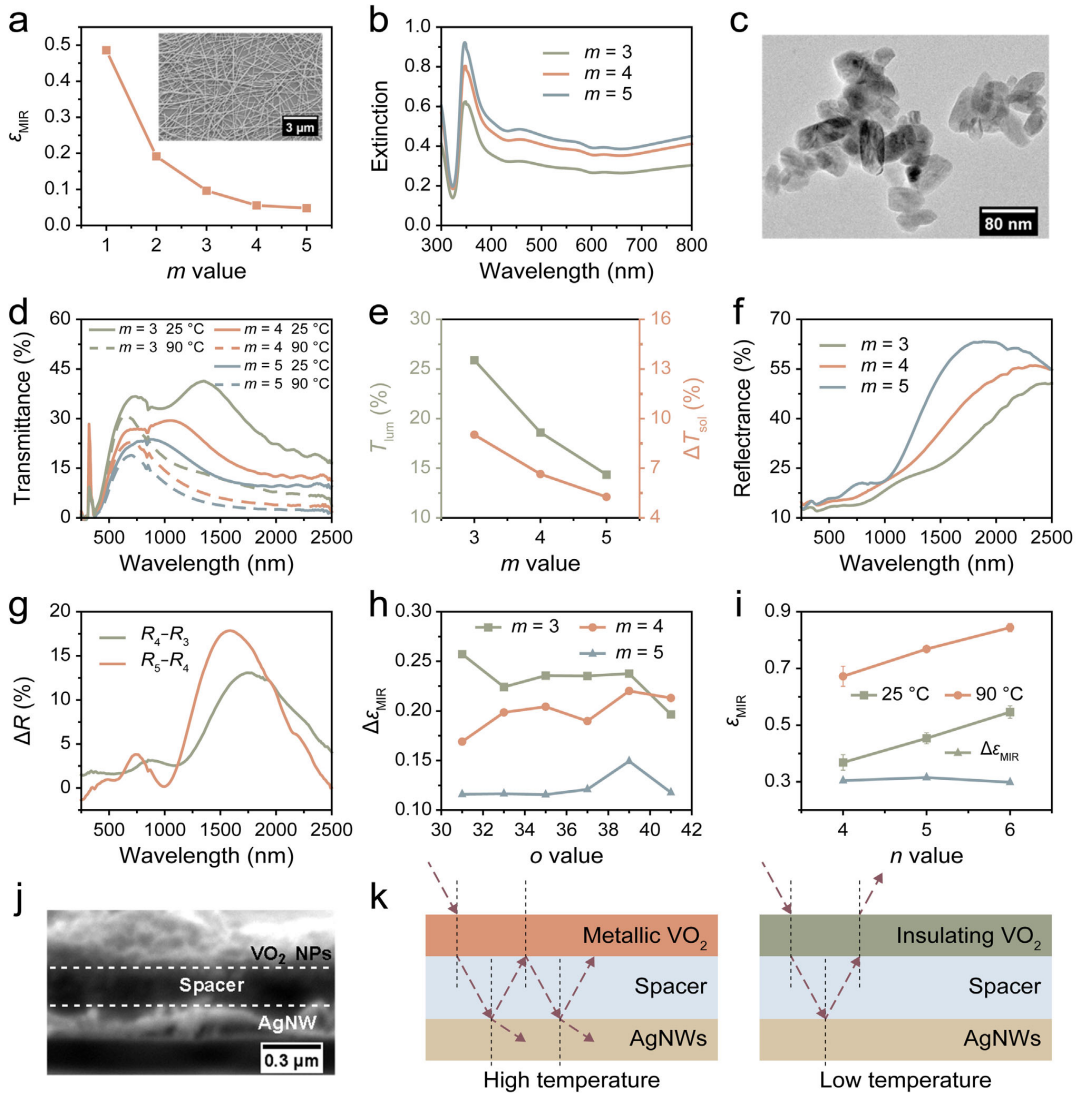
(a) The *ε*_MIR_ of the (PEI/AgNWs)_m_ coating with varying *m* values (insert: SEM image of the (PEI/AgNWs)_3_ structure). (b) Extinctions of the (PEI/AgNWs)_m_ coatings with varying *m*. (c) TEM image of spherical VO_2_ NPs. (d) Transmittance spectra of the glass/(PEI/AgNWs)_m_/(PDDA/PAA)_39_/(PDDA/PAA/PDDA/VO_2_)_5_ superstructures with varying *m* at 25°C and 90°C. (e) *T*_lum_ and Δ*T*_sol_ of the glass/(PEI/AgNWs)_m_/(PDDA/PAA)_39_/(PDDA/PAA/PDDA/VO_2_)_5_ superstructures with varying *m* values. (f) Reflectance spectra of the glass/(PEI/AgNWs)_m_/(PDDA/PAA)_39_/(PDDA/PAA/PDDA/VO_2_)_5_ superstructures with different *m* at 25°C. (g) Δ*R* observed when the *m* value increases from 3 to 4 and from 4 to 5. (h) Δ*ε*_MIR_ for the glass/(PEI/AgNWs)_m_/(PDDA/PAA)_o_/(PDDA/PAA/PDDA/VO_2_)_5_ superstructures with different *m* and *o* values. (i) The impact of the *n* values on *ε*_MIR_ regulation performance. (j) Cross‐sectional SEM image of the glass/(PEI/AgNWs)_3_/(PDDA/PAA)_39_/(PDDA/PAA/PDDA/VO_2_)_5_ superstructure. (k) Working mechanism of the F‐P cavity structure, illustrating the variation in *ε*_MIR_ at different temperatures.

The *ε*_MIR_ of the underlying coatings can be effectively controlled by varying the *m* value of the (PEI/AgNWs)_m_ low *ε*_MIR_ coating (Figure 2a). When *m* is ≥ 3, the *ε*_MIR_ is < 0.1, which is comparable to the *ε*_MIR_ of ITO‐coated glass. The insert in Figure 2a presents a scanning electron microscopy (SEM) image of the (PEI/AgNWs)_3_ structure, showing individual AgNWs (zeta potential: ‐33 mV) with diameters ranging from 40 to 60 nm and an aspect ratio of approximately 110. Figure 2b illustrates the extinction properties of the (PEI/AgNWs)_m_ coatings with different *m* values, demonstrating a consistent increase in extinction as *m* increases. Figure 2c depicts the transmission electron microscopy (TEM) image of the VO_2_ NPs (zeta potential: ‐76 mV), which exhibit a spherical morphology with a narrow size distribution and an average diameter of approximately 60 nm.

Figure 2d presents the transmittance spectra of the glass/(PEI/AgNWs)_m_/(PDDA/PAA)_39_/(PDDA/PAA/PDDA /VO_2_)_5_ superstructures with varying *m* values at 25°C and 90°C (with PDDA at 0.25 mg/mL and PAA at 2 mg/mL). It is evident that all the transmittance spectra exhibit interference effects, which diminish as *m* increases. Furthermore, the interference peaks in the transmittance spectra differ at 25°C and 90°C, likely due to VO_2_ having different n, k values at low and high temperatures. Additionally, the transmittance from the visible to the NIR range also decreases with increasing *m*. The calculated *T*_lum_ for the superstructures was found to be 25.9%, 18.6%, and 14.4% for *m* values of 3, 4, and 5, respectively (Figure 2e). A similar trend is observed for the solar modulation property: Δ*T*_sol_ decreases from 9.02% to 5.26% as the *m* value increases from 3 to 5 (Figure 2e).

Figure 2f displays the reflection spectra of the glass/(PEI/AgNWs)_m_/(PDDA/PAA)_39_/(PDDA/PAA/PDDA/VO_2_)_5_ superstructures with varying *m* values at 25°C. It is evident that the reflectance increases as the *m* value rises. To quantify this increase in reflectance with the addition of AgNWs layers, we calculated the difference in reflectance between the glass/(PEI/AgNWs)_3_/(PDDA/PAA)_39_/(PDDA/PAA/PDDA/VO_2_)_5_ sample and the glass/(PEI/AgNWs)_4_ /(PDDA/PAA)_39_/(PDDA/PAA/PDDA/VO_2_)_5_ sample, as well as the difference between the glass/(PEI/AgNWs)_4_/(PDDA/PAA)_39_/(PDDA/PAA/PDDA/VO_2_)_5_ sample and the glass/(PEI/AgNWs)_5_ /(PDDA/PAA)_39_ /(PDDA/PAA/PDDA/VO_2_)_5_ sample. These differences are graphically represented in Figure 2g. It can be observed that the reflectance difference (Δ*R*) is minimal for wavelengths ≤ 1200 nm or ≥ 2200 nm, while Δ*R* exhibits a sharp increase around ∼ 1600 nm. These spectrally selective properties may be attributed to intraband transitions of conduction electrons near the plasmon frequency [56, 57, 58]. Figures S3 and S4 present the transmittance and reflectance spectra of the glass/(PEI/AgNWs)_3_/(PDDA/PAA)_o_/(PDDA/PAA/PDDA/VO_2_)_5_ samples with varying *o* values at 25°C. The differences in transmittance and reflectance are negligible when changing the spacer thickness (*o* value), likely due to the transparent characteristics of the (PDDA/PAA)_o_ thin films.

The influence of the *m* and *o* values on *ε*_MIR_ regulation was investigated. Figure S5 illustrates the *ε*_MIR_ of the glass/(PEI/AgNWs)_m_/(PDDA/PAA)_o_/(PDDA/PAA/PDDA/VO_2_)_5_ superstructures with different *m* and *o* values. All the superstructures exhibit *ε*_MIR_ switching behavior. For instance, the glass/(PEI/AgNWs)_3_/(PDDA/PAA)_o_ /(PDDA/PAA/PDDA/VO_2_)_5_ superstructures demonstrate an *ε*_MIR_ of approximately 0.4 at 25°C. This value increases to around 0.7 when the temperature rises to 90°C, resulting in a Δ*ε*_MIR_ of approximately 0.3. This behavior may be attributed to the low *ε*_MIR_ coating/spacer/VO_2_ configuration forming a F‐P resonator [59]. Such a resonator exhibits low resonance for *ε*_MIR_ at lower temperatures, but displays pronounced F‐P resonance behavior, resulting in significantly enhanced *ε*_MIR_ absorption at higher temperatures due to the insulator‐to‐metal transition of VO_2_ [60].

Both *ε*_MIR_ values at 25°C and 90°C decrease with increasing *m*, which can be attributed to the critical role of the AgNWs underlayer in determining *ε*_MIR_. However, *ε*_MIR_ remains nearly constant with increasing *o* values. The best regulation performance of *ε*_MIR_ was observed when *m* = 3, with Δ*ε*_MIR_ decreasing as the *m* value increases (Figure 2h). Figure 2i illustrates the effect of the number of top VO_2_ layers (*n* value) on the *ε*_MIR_ regulation behavior of the ITO/(PDDA/PAA)_28_/(PDDA/PAA/PDDA/VO_2_)_n_ RC windows. It can be observed that the LbL‐assembled RC windows exhibit optimal *ε*_MIR_ regulation performance when the *n* value is 5.

Figure 2j presents a cross‐sectional SEM image of a glass/(PEI/AgNWs)_3_/(PDDA/PAA)_39_/(PDDA/PAA/PDDA /VO_2_)_5_ RC window. In this structure, the top block (PDDA/PAA/PDDA/VO_2_)_5_ and the bottom block (PEI/AgNWs)_3_ are separated by a multilayered (PDDA/PAA)_39_ spacer.

The working mechanism of this F‐P cavity is illustrated in Figure 2k. This structure consists of two “mirrors” separated by a lossless spacer. As depicted in Figure 2k, temperature variations induce a phase transition in VO_2_, resulting in tunable *ε*_MIR_. At elevated temperatures, VO_2_ transitions to a metallic state, functioning as the top mirror of the F‐P resonator. This configuration enhances *ε*_MIR_, facilitating effective RC (Figure 2k, left). Conversely, at lower temperatures, VO_2_ becomes insulating and fails to provide significant optical enhancement (Figure 2k, right). In this insulating state, both the VO_2_ and the spacer are transparent in the MIR range, leading to low *ε*_MIR_ due to the underlying AgNWs, which suppresses the RC effect.

**FIGURE 3:**
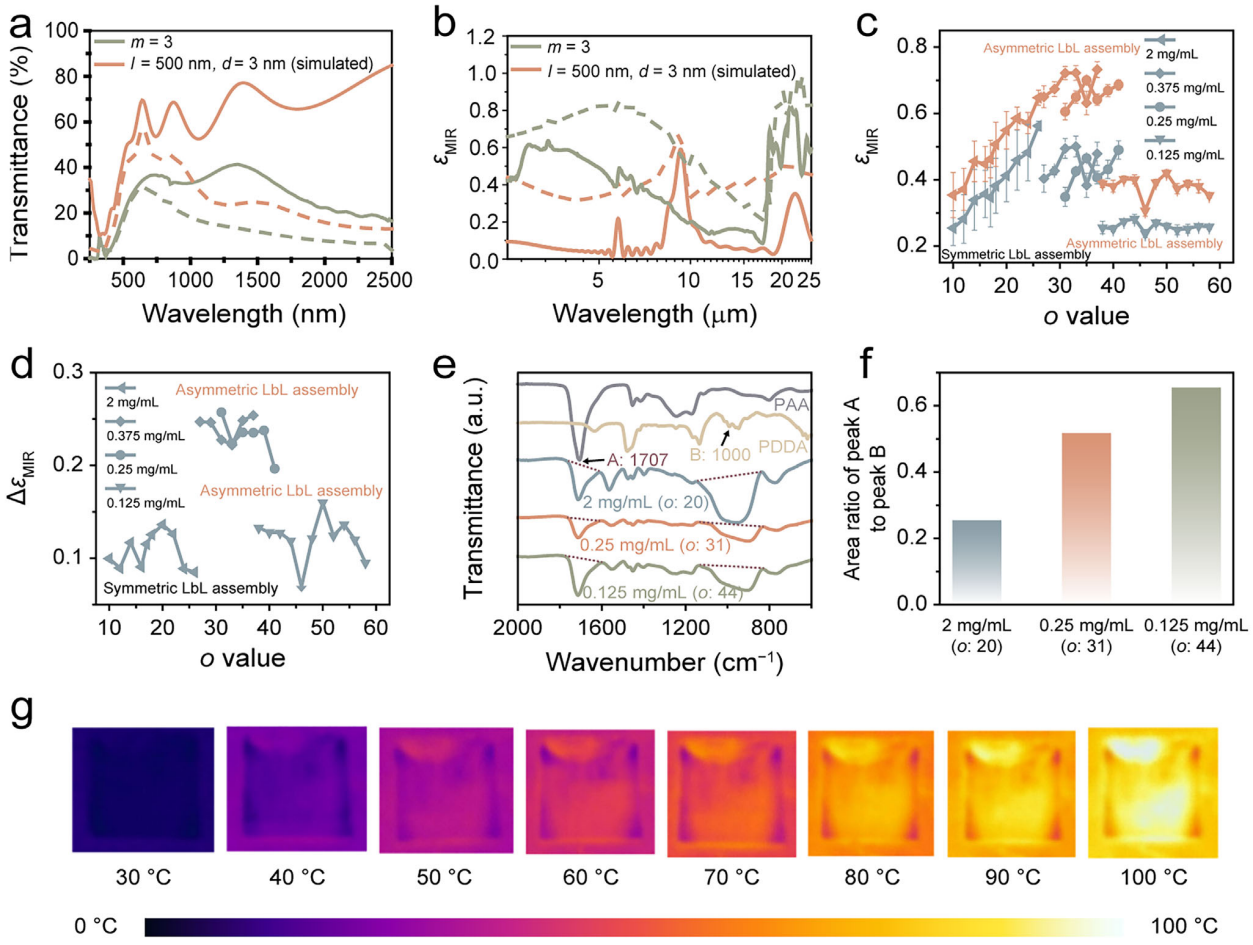
(a) Comparison of simulated UV‐vis‐NIR transmittance spectra for the superstructure (*l* = 500 nm, *d* = 3 nm) and the measured transmittance spectra of the glass/(PEI/AgNWs)_3_/(PDDA/PAA)_39_/(PDDA/PAA/PDDA/VO_2_)_5_ superstructure at 25°C (solid line) and 90°C (dashed line). (b) Comparison of simulated *ε*_MIR_ for the superstructure (*l* = 500 nm, *d* = 3 nm) and the measured *ε*_MIR_ for the glass/(PEI/AgNWs)_3_/(PDDA/PAA)_39_/(PDDA/PAA/PDDA/VO_2_)_5_ superstructure at the same temperatures (25°C as solid line and 90°C as dashed line). (c) *ε*_MIR_ values for the glass/(PEI/AgNWs)_3_/(PDDA/PAA)_o_/(PDDA/PAA/PDDA/VO_2_)_5_ superstructures, prepared with varying PDDA concentrations (2, 0.375, 0.25, and 0.125 mg/mL) at 25°C (blue) and 90°C (red). (d) The Δ*ε*_MIR_ of the superstructures calculated from (c). (e) FTIR spectra of PAA, PDDA, and the (PDDA/PAA)_o_ spacers at different PDDA concentrations: (PDDA/PAA)_20_ (2 mg/mL), (PDDA/PAA)_31_ (0.25 mg/mL), and (PDDA/PAA)_44_ (0.125 mg/mL). (f) The area ratio of peak A (C═O peak of PAA) to peak B (C─N^+^ peak of PDDA) for different (PDDA/PAA)_o_ spacers. (g) IR camera images of the glass/(PEI/AgNWs)_3_/(PDDA/PAA)_39_/(PDDA/PAA/PDDA/VO_2_)_5_ sample, with a background *ε*_MIR_ of 0.5, were captured at various temperatures.

Figure 3a displays the simulated UV‐vis‐NIR transmittance spectra for the superstructure (*l* = 500 nm, *d* = 3 nm) and the measured transmittance spectra of the glass/(PEI/AgNWs)_3_/(PDDA/PAA)_39_/(PDDA/PAA/PDDA/VO_2_)_5_ superstructure. Figure S6 presents the simulated UV‐vis‐NIR transmittance spectra for the superstructures with varying interlayer spacers (*d*) between VO_2_ NPs layers, as well as for the superstructure with a continuous VO_2_ thin film. Additionally, it includes the measured transmittance spectra for the glass/(PEI/AgNWs)_m_/(PDDA/PAA)_39_/(PDDA/PAA/PDDA/VO_2_)_5_ superstructures with varying *m*. Consistent with the measured transmittance spectra, all simulated spectra exhibit interference effects. Notably, an interlayer spacer of 3 nm between VO_2_ NPs enhances the *T*_lum_ at both low (solid line) and high (dashed line) temperatures compared to the superstructure without an interlayer spacer (*d* = 0 nm) and the superstructure with a continuous VO_2_ thin film (60 nm) (*d =* 3 nm: 48.6%; *d =* 0 nm: 37.1%; continuous thin film: 33.7%) (Figure S6 and Table S1). This enhancement is likely due to the interlayer spacer (*d*) facilitating a better distribution of light reflection and absorption, thereby increasing *T*_lum_.

Furthermore, the Δ*T*_sol_ of the superstructure with an interlayer spacer (*d* = 3 nm) shows significant improvement compared to both the superstructure without an interlayer spacer (*d* = 0 nm) and the superstructure with a continuous VO_2_ thin film (60 nm) (*d =* 3 nm: 19.2%; *d =* 0 nm: 11.4%; continuous thin film: 9.69%) (Table S1). This enhancement may stem from the additional interference induced by the interlayer spacer (*d*), in conjunction with the interference effects present in the multilayered superstructure (low *ε*_MIR_ coating/spacer/VO_2_ layers).

Figure 3b presents the simulated *ε*_MIR_ for the superstructure (*l* = 500 nm, *d* = 3 nm) and the measured *ε*_MIR_ for the glass/(PEI/AgNWs)_3_/(PDDA/PAA)_39_/(PDDA/PAA/PDDA/VO_2_)_5_ superstructure. Figure S7 shows the simulated *ε*_MIR_ for superstructures with varying interlayer spacers (*d*) between VO_2_ NPs layers, as well as for the superstructure with a continuous VO_2_ thin film. It also includes the measured *ε*_MIR_ for the glass/(PEI/AgNWs)_3_/(PDDA/PAA)_39_/(PDDA/PAA/PDDA/VO_2_)_5_ superstructure. Notably, all IR spectra at 90°C reveal a higher *ε*_MIR_ of approximately 0.3 compared to its value at 25°C (Figure 3b; Figure S7). The simulations indicate that the superstructure with an interlayer spacer of 3 nm between VO_2_ NP layers exhibits similar *ε*_MIR_ switching performance to that of the superstructure without an interlayer spacer (0 nm) and the superstructure with a continuous VO_2_ thin film (60 nm) (Figure S7). The differences between the experimental and simulated results for both transmittance and *ε*_MIR_ may be attributed to morphological irregularities. In the simulations, the components were treated as ideally assembled, and the surfaces were considered atomically smooth. However, in experiments, it is challenging to achieve ideally assembled thin films.

The influence of the chemical composition of the (PDDA/PAA)_o_ spacers on the regulation of *ε*_MIR_ was investigated. The chemical composition of the (PDDA/PAA)_o_ spacers was adjusted by varying the concentration of PDDA while maintaining a constant PAA concentration of 2 mg/mL, as PAA exhibits low absorption between 8 and 14 µm (1250 – 714 cm^−1^) (Figure 3e). Figure 3c illustrates the *ε*_MIR_ values for the glass/(PEI/AgNWs)_3_/(PDDA/PAA)_o_ /(PDDA/PAA/PDDA/VO_2_)_5_ superstructures prepared with varying PDDA concentrations (2, 0.375, 0.25, and 0.125 mg/mL) at 25°C and 90°C. It is evident that as the PDDA concentration increases, the number of PDDA/PAA layer pairs (*o* value) required for optimal *ε*_MIR_ regulation decreases. This trend may be attributed to higher PDDA concentrations promoting a more efficient formation of the LbL‐assembled spacer. When the concentration of PDDA is equal to that of PAA, the LbL assembly can be classified as symmetric. Conversely, when the PDDA concentration is lower than that of PAA, the assembly is categorized as asymmetric (Figure 3c).

The optimal *ε*_MIR_ regulation performance of the glass/(PEI/AgNWs)_3_/(PDDA/PAA)_o_/(PDDA/PAA/PDDA/VO_2_)_5_ superstructures is observed at PDDA concentrations of 0.375 and 0.25 mg/mL (Figure 3c). This performance can be attributed to the unique composition of the (PDDA/PAA)_o_ spacers, where PAA contains IR‐transmitting bonds (O─H, C═O, C─H, and C─C) within the range of 1250–714 cm^−1^, while exhibiting minimal IR absorption from the C─O bond at approximately 1240 and 1170 cm^−1^ (Figure 3e). Furthermore, at lower concentrations, PDDA acts as the minor component in the (PDDA/PAA)_o_ spacers. The C─H stretching vibrations of ─CH_3_ and ─CH_2_ groups, C─C skeletal vibrations, and out‐of‐plane bending of C─H, along with the asymmetric and symmetric C─H bending of ─CH_3_ (N^+^─CH_3_), contribute to efficient IR transmission. However, the IR absorption associated with the C─N stretching vibrations (N^+^─(CH_3_)_2_) is significantly reduced within the same spectral range (1250 – 714 cm^−1^). The Δ*ε*_MIR_ significantly decreases at PDDA concentrations of 2 and 0.125 mg/mL. At 2 mg/mL, the Δ*ε*_MIR_ of the glass/(PEI/AgNWs)_3_/(PDDA/PAA)_o_/(PDDA/PAA/PDDA/VO_2_)_5_ superstructures is suppressed due to strong PDDA absorption (Figure 3c,d). As the *o* value increases, the *ε*_MIR_ values at both 25°C and 90°C rise simultaneously, resulting in a Δ*ε*_MIR_ of approximately 0.1 (Figure 3c,d). Conversely, at a PDDA concentration of 0.125 mg/mL, the Δ*ε*_MIR_ is also observed to be nearly 0.1. This diminished Δ*ε*_MIR_ may be attributed to the suppression of *ε*_MIR_ values at both 25°C and 90°C (Figure 3c,d).

Figure 3e displays the FTIR spectra of PAA, PDDA, and the (PDDA/PAA)_o_ spacers at different PDDA concentrations: (PDDA/PAA)_20_ (2 mg/mL), (PDDA/PAA)_31_ (0.25 mg/mL), and (PDDA/PAA)_44_ (0.125 mg/mL). It can be concluded that PAA exhibits a strong C═O peak at 1707 cm^−1^ (Peak A), while PDDA displays a distinct C─N^+^ peak at approximately 1000 cm^−1^ (Peak B). All spacers exhibit these two peaks in their FTIR spectra (Figure 3e). The area ratio of Peak A to Peak B is presented in Figure 3f. As the PDDA concentration decreases from 2 to 0.125 mg/mL, the area ratio of Peak A (C═O peak of PAA) to Peak B (C─N^+^ peak of PDDA) increases from 0.254 to 0.655. This trend confirms that varying the concentration of PDDA can tune the chemical composition of the spacer, ultimately enhancing the *ε*_MIR_ regulation performance.

Figure 3g and Figure S8 present IR camera images of the glass/(PEI/AgNWs)_3_/(PDDA/PAA)_39_ /(PDDA/PAA/PDDA/VO_2_)_5_ sample (Figure 3g) and the glass/(PDDA/PAA)_39_/(PDDA/PAA/PDDA/VO_2_)_5_ sample (Figure S8), both evaluated at a background *ε*_MIR_ of 0.5 across different temperatures. From 30°C to 60°C, the glass/(PEI/AgNWs)_3_/(PDDA/PAA)_39_/(PDDA/PAA/PDDA/VO_2_)_5_ sample exhibits darker colors compared to the background, indicating a lower *ε*_MIR_ (0.4 vs. 0.5), as a higher *ε*_MIR_ corresponds to increased thermal radiation intensity. When the temperature exceeds 70°C, the RC window displays a brighter color than the background, reflecting a higher *ε*_MIR_ (0.7 vs. 0.5) (Figure 3g). In contrast, the glass/(PDDA/PAA)_39_/(PDDA/PAA/PDDA/VO_2_)_5_ sample consistently appears brighter than the background, demonstrating its lack of *ε*_MIR_ regulation capability (Figure S8).

**FIGURE 4:**
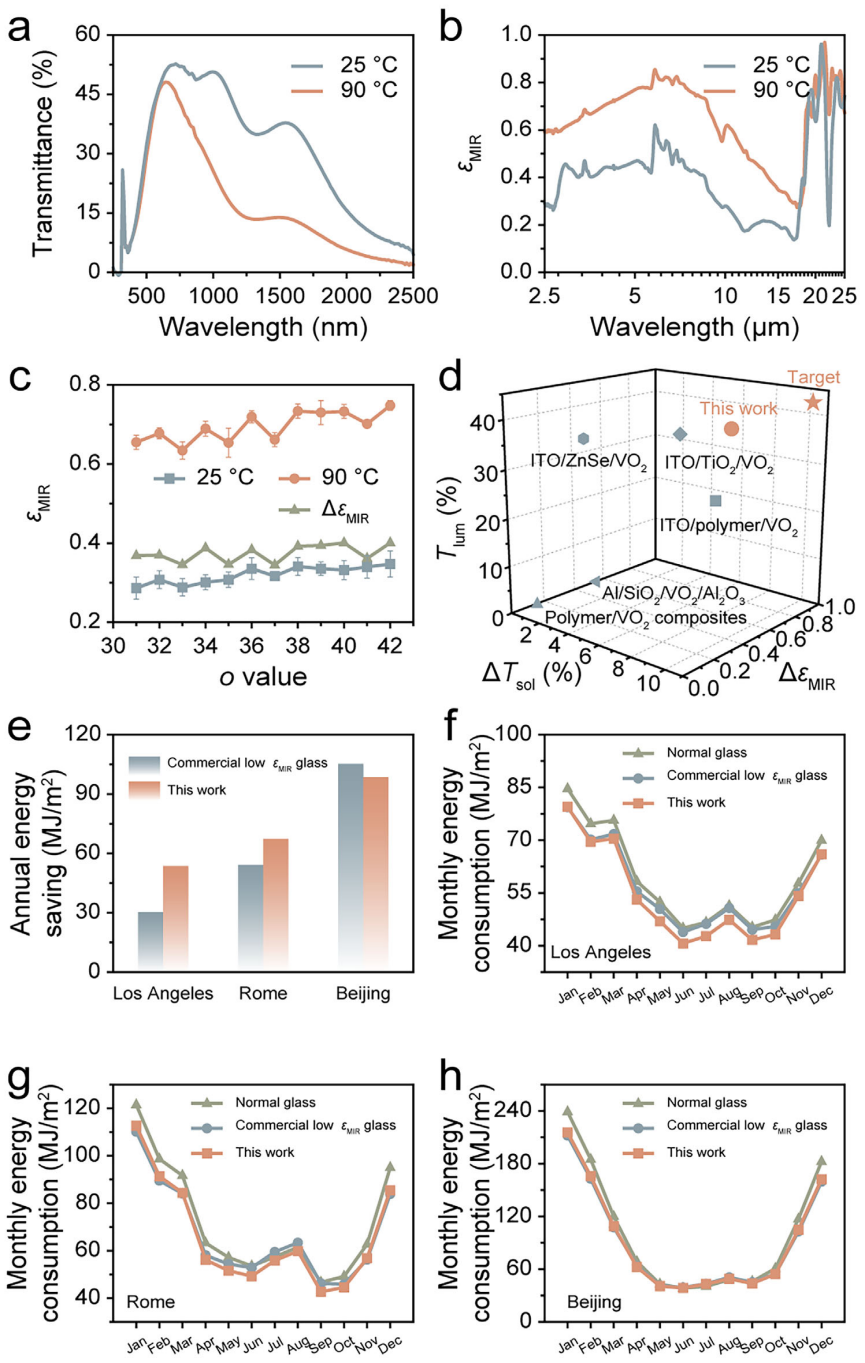
(a) Transmittance spectra of the glass/ITO/(PDDA/PAA)_31_/(PDDA/PAA/PDDA/VO_2_)_5_ superstructure at 25°C and 90°C. (b) *ε*_MIR_ of the glass/ITO/(PDDA/PAA)_37_/(PDDA/PAA/PDDA/VO_2_)_5_ superstructure at 25°C and 90°C. (c) *ε*_MIR_ regulation performance of the ITO/(PDDA/PAA)_o_/(PDDA/PAA/PDDA/VO_2_)_5_ samples with different *o* values. (d) Comparison of *T*_lum_, Δ*T*_sol_, and Δ*ε*_MIR_ for various reported windows, including ITO/polymer/VO_2_ [14], polymer/VO_2_ composites [54], ITO/TiO_2_/VO_2_ [24], ITO/ZnSe/VO_2_ [55], Al/SiO_2_/VO_2_/Al_2_O_3_ [25], and our LbL‐assembled RC window. (e) Annual energy‐saving performance of commercial low *ε*_MIR_ glass compared to the LbL‐assembled RC window across various cities, including Los Angeles, Rome, and Beijing. (f) Monthly energy consumption for normal glass, commercial low *ε*_MIR_ glass, and the LbL‐assembled RC window in Los Angeles. (g) Monthly energy consumption for normal glass, commercial low *ε*_MIR_ glass, and the LbL‐assembled RC window in Rome. (h) Monthly energy consumption for normal glass, commercial low *ε*_MIR_ glass, and the LbL‐assembled RC window in Beijing.

After establishing the optimal parameters for fabricating the F‐P cavity, the cavity was also constructed using ITO as the low *ε*_MIR_ coating. Figure 4a displays the transmittance spectra of the glass/ITO/(PDDA/PAA)_31_/(PDDA/PAA/PDDA/VO_2_)_5_ superstructure. The *T*_lum_ and Δ*T*_sol_ values were found to be 41% and 10.1% for the glass/ITO/(PDDA/PAA)_31_/(PDDA/PAA/PDDA/VO_2_)_5_ superstructure (Figure S9). The low transmittance observed at wavelengths longer than 1500 nm for the glass/ITO/(PDDA/PAA)_31_ /(PDDA/PAA/PDDA/VO_2_)_5_ superstructure can be attributed to the strong NIR blocking properties of the ITO coating.

The ITO/(PDDA/PAA)_o_/(PDDA/PAA/PDDA/VO_2_)_5_ superstructures exhibit similar *ε*_MIR_ switching behavior, as further corroborated by IR spectra (Figure 4b). The optimal Δ*ε*_MIR_ of these superstructures is approximately 0.4 (Figure 4c). In contrast, the glass/(PDDA/PAA)_39_/(PDDA/PAA/PDDA/VO_2_)_5_ superstructure exhibits a *T*_lum_ of 45.4% and a Δ*T*_sol_ of 14.2%, but shows negligible *ε*_MIR_ switching performance (Figures S10–S13). This indicates that the design of the low *ε*_MIR_ coating/spacer/VO_2_ layers is critical for achieving a favorable Δ*ε*_MIR_.

The solar modulation and RC regulation performances of our LbL‐assembled RC window have been compared with previously reported windows. As illustrated in Figure 4d, the LbL‐assembled RC window demonstrates significantly higher *T*_lum_ and Δ*T*_sol_, as well as higher or comparable Δ*ε*_MIR_ values compared to other reported windows, including ITO/polymer/VO_2_ [14], polymer/VO_2_ composites [54], ITO/TiO_2_/VO_2_ [24], ITO/ZnSe/VO_2_ [55], and Al/SiO_2_/VO_2_/Al_2_O_3_ [25]. The superior solar modulation and RC regulation performances of our LbL‐assembled RC window can be attributed to the LbL assembly technique, which enables precise control over both the quantity and spatial arrangement of functional components within the structure.

### 2.2 Energy Saving Simulation

The annual energy‐saving performance of the LbL‐assembled RC window was simulated alongside that of commercial low *ε*_MIR_ glass in three cities: Los Angeles, Rome, and Beijing, with normal glass serving as the baseline (Figure 4e). The LbL‐assembled RC window exhibits a 77.5% and 24.6% improvement in annual energy‐saving performance compared to the commercial low *ε*_MIR_ glass in Los Angeles and Rome, respectively. Additionally, in Beijing, the LbL‐assembled RC window shows comparable energy‐saving performance to the commercial low *ε*_MIR_ glass (commercial low *ε*_MIR_ glass: 8.9%, LbL‐assembled RC window: 8.3%).

Figure 4f–h illustrates the monthly energy consumption for the three types of windows in Los Angeles, Rome, and Beijing. Throughout the year, the LbL‐assembled RC window consistently saves more energy than normal glass in all three cities. It outperforms commercial low *ε*_MIR_ glass for nearly the entire year (from February to November) in Los Angeles and demonstrates superior energy‐saving performance for over half the year (from April to October) in both Rome and Beijing.

Consequently, during the hotter months, the LbL‐assembled RC window provides greater energy savings compared to both normal glass and commercial low *ε*_MIR_ glass. In the colder months, it shows comparable energy‐saving performance to commercial low *ε*_MIR_ glass while outperforming normal glass. These energy consumption simulations confirm the excellent energy‐saving capability of the LbL‐assembled RC window across different climate zones.

## 3 Conclusion

In summary, a thermochromic F‐P superstructure has been designed and fabricated using a unique spectral‐selective LbL assembly technique, enabling year‐round energy savings across various climate zones. By thoughtfully selecting MIR‐transparent polycations and polyanions and employing an asymmetric assembly approach, these superstructures, represented as ITO/(PDDA/PAA)_o_/(PDDA/PAA/PDDA/VO_2_)_n_ (*o* or *n* = 1, 2, 3…), have demonstrated high optical performance, achieving a high *T*_lum_ of 41%, a Δ*T*_sol_ of 10.1%, and a Δ*ε*_MIR_ of 0.4, outperforming existing state‐of‐the‐art solutions. In building energy simulations, the LbL‐assembled window demonstrates superior annual energy‐saving performance compared to commercial low *ε*_MIR_ glass across different climate zones. The ease of fabrication, combined with the excellent energy‐saving capabilities of this window, significantly contributes to the overarching goal of achieving global carbon neutrality.

## 4 Experimental Section

### 4.1 Materials

Pure VO_2_ was obtained from Ji‐Cheng (China). Ethanol absolute (99.9%) was sourced from Merck (Germany). Sodium chloride, silver nitrate (AgNO_3_), glycerol, poly(vinylpyrrolidone) (PVP, Mw ≈ 40,000), poly(ethyleneimine) (PEI, Mn ≈ 60,000 g/mol), poly(acrylic acid) (PAA, M_V_ ≈ 450,000 g/mol), and poly(diallyldimethylammonium chloride) (PDDA, M_W_ ≈ 200,000–350,000 g/mol, 20 wt.% in H_2_O) were purchased from Sigma‐Aldrich. All chemicals were used as received. ITO‐coated glass slides were obtained from Winteck Technology. Aqueous solutions were prepared using ultrapure water (resistivity = 18.2 MΩ·cm, Milli‐Q Gradient system).

PEI solutions were prepared by dissolving the polymer in ultrapure water at a concentration of 5 mg/mL. PAA solutions were obtained by dissolving the polymer in ultrapure water at a concentration of 2 mg/mL, with the pH adjusted to 5.0‐6.0 using a 1 m NaOH solution. PDDA solutions were prepared by diluting the stock PDDA solution with ultrapure water to achieve concentrations of 2, 0.375, 0.25, and 0.125 mg/mL. VO_2_ suspensions were created by dispersing VO_2_ powder in ethanol at a concentration of 10 mg/mL, using sonication for over 5 h. The upper portion of these suspensions was utilized for film preparation.

### 4.2 Synthesis of AgNWs

AgNWs were synthesized following established procedures [61]. Briefly, 7.04 g of PVP was dissolved in 228 mL of glycerol at 110°C under continuous stirring. After the solution cooled to room temperature, 1.90 g of AgNO_3_ was added, followed by a mixture of 80 mg NaCl, 0.60 mL of ultrapure water, and 12 mL of glycerol. The entire system was then heated from room temperature to 210°C within 40 min while stirring. Once at 210°C, the heating was discontinued, and the solution was cooled to 110°C, after which 240 mL of ultrapure water was added. The system was allowed to cool further to room temperature and was maintained stable for one week. The sediment formed at the bottom of the flask was carefully collected. The resulting AgNWs were washed with ethanol three times by centrifugation at 6000 rpm for 10 min. Finally, the product was suspended in 30 mL of ethanol, yielding an upper solution with a concentration of approximately 7 mg/mL, which was used for film fabrication.

### 4.3 Preparation of Low *ε*_MIR_ Coating Based on AgNWs

Glass slides were first activated by UV ozone treatment for 15 min. Following activation, the slides were dipped in a PEI aqueous solution for 10 min, rinsed with ultrapure water (3 × 1 min), and then dried with compressed argon. The PEI‐coated slides were subsequently immersed into the AgNWs suspension at a dipping speed of 100 mm/min until reaching the bottom of the vial. After remaining undisturbed for 2 s, the slides were lifted out of the suspension at the same speed, allowing excess ethanol to evaporate. This dipping process was repeated five times.

Next, the AgNWs‐loaded slides were coated with a second layer of PEI, followed by another layer of AgNWs under the same conditions. These deposition steps could be repeated multiple times. The resulting low *ε*_MIR_ coatings are denoted as (PEI/AgNWs)_m_, where *m* represents the number of layer repetitions (*m* = 1, 2, 3, …).

### 4.4 Preparation of the Coating With Regulated *ε*_MIR_ and Solar Modulation

To create a window with regulated *ε*_MIR_ and solar modulation, structures of the form (PDDA/PAA)_o_/(PDDA/PAA/PDDA/VO_2_)_n_ (where *o* and *n* can be 1, 2, 3, …) were applied on top of the low *ε*_MIR_ coating (PEI/AgNWs)_m_ (where *m* can also be 1, 2, 3, …). In this configuration, the top multilayers (PDDA/PAA/PDDA/VO_2_)_n_ are responsible for solar modulation, while the (PDDA/PAA)_o_ multilayers are utilized to adjust the spacer thickness between the top VO_2_ multilayers and the underlying low *ε*_MIR_ coating.

The PDDA and PAA layers were applied by dipping the substrate in their respective solutions for 5 min, followed by rinsing with ultrapure water (3 × 1 min). The VO_2_ was coated using a method similar to that employed for the AgNWs. The substrate, with a top PDDA layer, was immersed in a pure VO_2_ suspension at a dipping speed of 100 mm/min until reaching the bottom of the vial. After remaining undisturbed for 2 s, the substrate was lifted out of the suspension at the same speed, allowing excess ethanol to evaporate. This dipping process was repeated twice before applying the next layer of PDDA.

ITO‐coated glass slides were activated by exposure to UV ozone for 15 min. Following this activation, the spacer and VO_2_ multilayers were coated on the slides using the same procedures as described above.

### 4.5 Characterizations

The integrated *ε*_MIR_ of the samples was measured using a dual‐band emissivity measuring device (IR‐2, Shanghai Chengbo Photoelectric Technology) equipped with a heating stage. Top‐view and cross‐sectional images were obtained using Scanning Electron Microscopy (JEOL 6700F).

IR spectra were recorded with a Perkin Elmer Frontier spectrometer. UV‐Vis‐NIR transmittance measurements were conducted using a Lambda 950 spectrophotometer, with air as the reference. UV‐vis absorbance was measured using a Cary 5000 spectrophotometer. Additionally, IR images were captured using an IR camera (C8940).

The integrated luminous transmittance (*T*_lum_) in the range of 380–780 nm, IR transmittance (*T*_IR_) from 780–2500 nm, and solar transmittance (*T*_sol_) from 250–2500 nm were calculated using the following Equation (1):

(1) $$\begin{array}{c} T_{\mathrm{lum}/\mathrm{IR}/\mathrm{sol}}=\int \varphi_{\mathrm{lum}/\mathrm{IR}/\mathrm{sol}}\left(\lambda \right)T\left(\lambda \right)d\left(\lambda \right)/\int \varphi_{\mathrm{lum}/\mathrm{IR}/\mathrm{sol}}\left(\lambda \right)d\left(\lambda \right) \end{array}$$

where *T*(λ) is the spectral transmittance, *φ*_lum_ (λ) represents the standard luminous efficiency function for photopic vision in the wavelength range of 380 and 780 nm, *φ*_IR_ (λ) and *φ*_sol_ (λ) denote the IR/solar irradiance spectrum distribution for air mass 1.5 (corresponding to the sun standing 37° above the horizon with 1.5 atmospheric thickness that corresponds to a solar zenith angle of 48.2). Δ*T*_lum/IR/sol_ can be obtained by Δ*T*_lum/IR/sol_ = *T*_lum/IR/sol, low temperature_—*T*_lum/IR/sol, high temperature_.

### 4.6 Optical Simulation

A finite‐difference time‐domain (FDTD) method was employed to simulate the optical transmittance and thermal emissivity of the LbL‐assembled RC window. The simulation was carried out using a three‐dimensional model with periodic boundary conditions applied in the x‐ and y‐directions, and perfectly matched layer (PML) boundary conditions in the z‐direction to absorb outgoing waves. A uniform square mesh with a spatial resolution of 2 nm was used throughout the simulation domain to ensure numerical accuracy and to capture subwavelength features in the nanostructure. The structure was illuminated along the z‐direction by a normally incident broadband plane wave source, spanning the desired spectral range for both solar and IR analysis. The structure was illuminated along the z‐direction by a broadband white light source with a normal incidence.

### 4.7 Energy‐Saving Simulation for LbL‐Assembled RC Windows

The HVAC energy consumption of LbL‐assembled RC windows was simulated using EnergyPlus software. For comparison, normal glass and commercial low *ε*_MIR_ glass were utilized, with their respective optical properties detailed in Table S2. The building area was set at 48 m^2^, and the window opening area was defined as 24 m^2^, resulting in a gross window‐to‐wall ratio of 31.75%. Climate data from Los Angeles, Rome, and Beijing were selected as the locations for the simulation. The energy consumption of LbL‐assembled RC windows was subsequently compared with that of normal glass and commercial low *ε*_MIR_ glass to quantify potential energy savings.

## Conflicts of Interest

The authors declare no conflicts of interest.

## Supporting information




**Supporting File**: advs74461‐sup‐0001‐SuppMat.docx.

## Data Availability

The data that support the findings of this study are available from the corresponding author upon reasonable request.
