# Supplementary material for: Dual‐Band Modulation of Emissivity and Solar Transmittance Through Chemical Composition‐Tuned Asymmetric Layer‐by‐Layer Assembly
Source: Adv Sci (Weinh). 2026 Feb 20;13(24):e74461. doi: 10.1002/advs.74461 (PMC13116205; doi:10.1002/advs.74461)
Supplement: Supplementary file 1 — Supporting File: advs74461‐sup‐0001‐SuppMat.docx. [file ADVS-13-e74461-s001.docx]

Dual-Band Modulation of Emissivity and Solar Transmittance Through Chemical Composition-Tuned Asymmetric Layer-by-Layer Assembly

Hebing Hu,* Zhengui Zhou, Guanya Wang, Xiaofei Li, Yun Meng,* Tao Xu, Xiao’e Jia, Hao Yu, Yuhan Wang, Jiarui Wang, Yi Long*


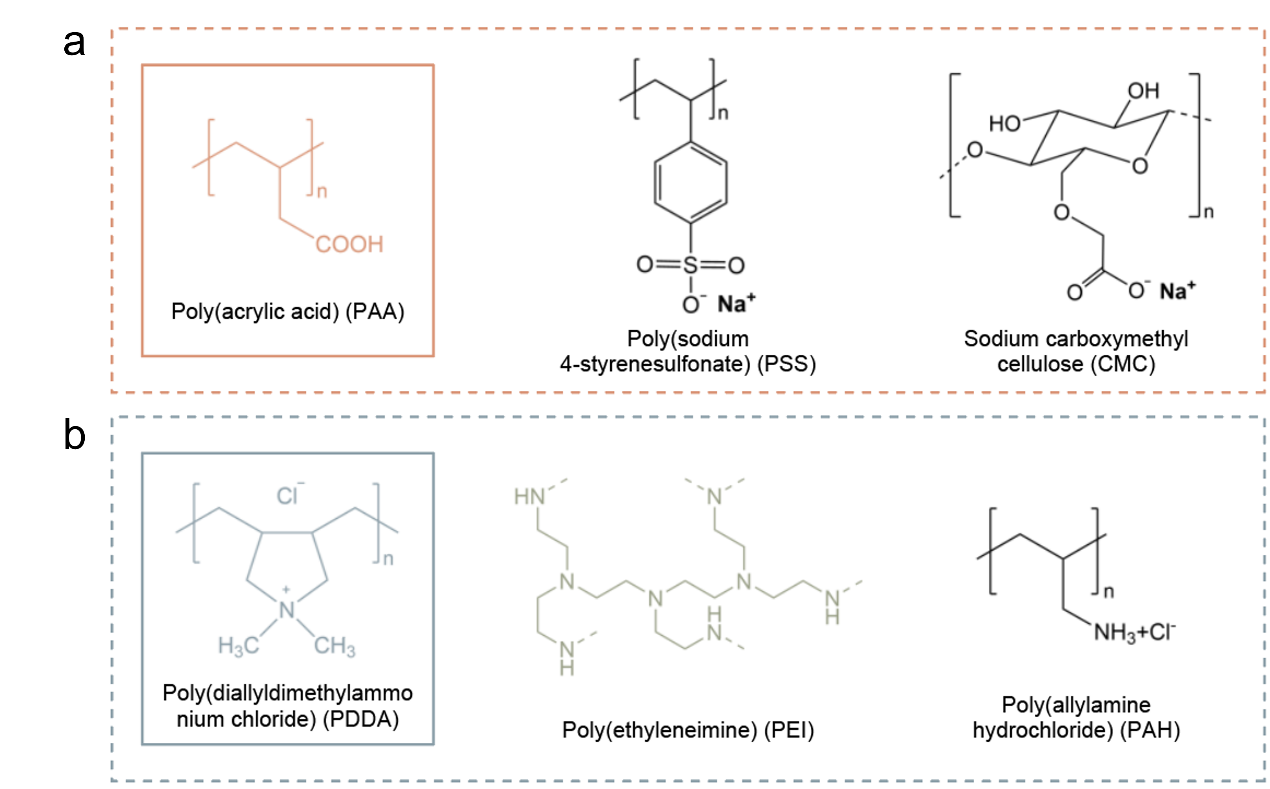


Figure S1. (a) Molecular structures of commonly used polyanions for LbL assembly. (b) Molecular structures of commonly used polycations for LbL assembly.


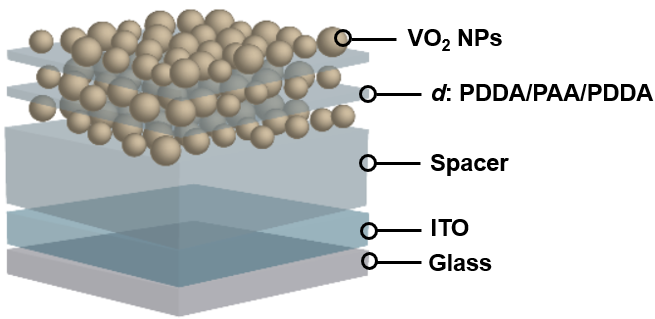


Figure S2. Schematic representation of the F-P cavity structure, featuring an ITO low *ε*_MIR_ coating, a pacer, and VO_2_ multilayers.


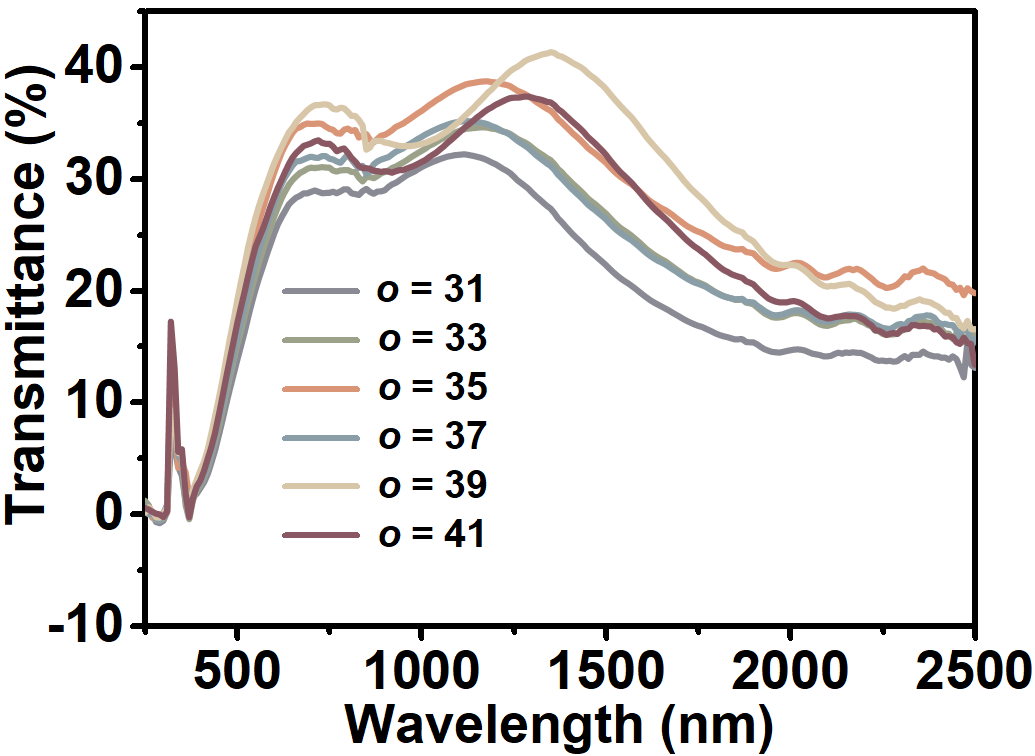


Figure S3. Transmittance spectra of the glass/(PEI/AgNWs)_3_/(PDDA/PAA)_o_

/(PDDA/PAA/PDDA/VO_2_)_5_ superstructures with varying *o* values at 25 ^o^C.


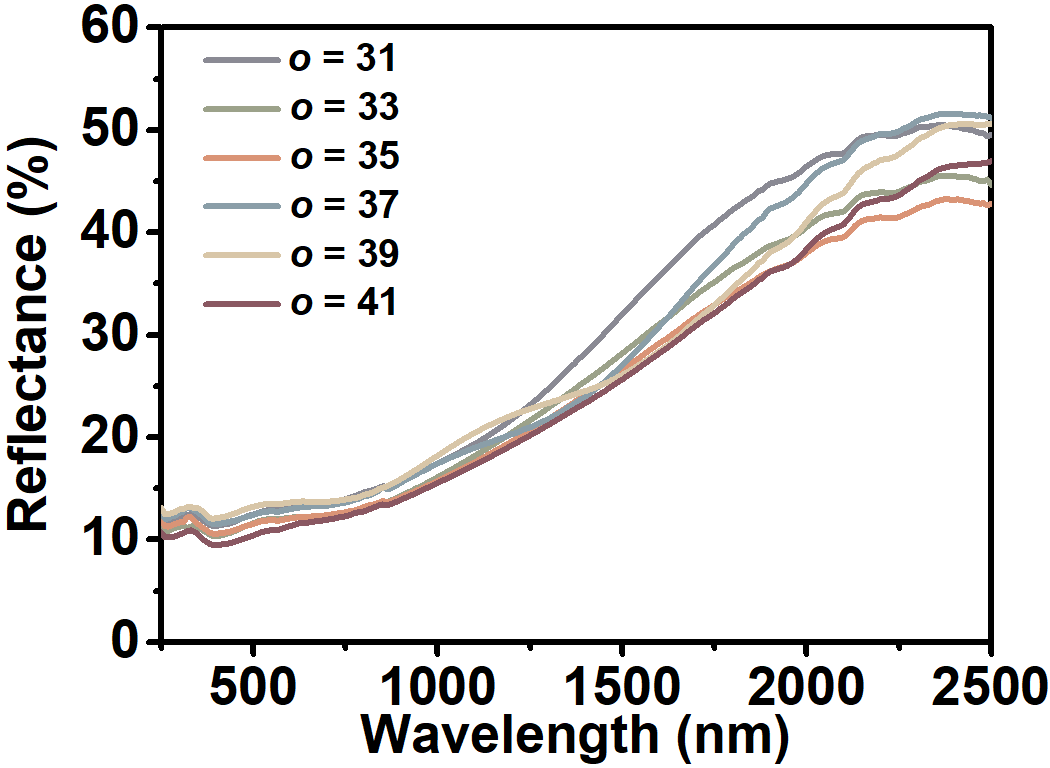


Figure S4. Reflectance spectra of the glass/(PEI/AgNWs)_3_/(PDDA/PAA)_o_

/(PDDA/PAA/PDDA/VO_2_)_5_ superstructures with varying *o* values at 25 ^o^C.


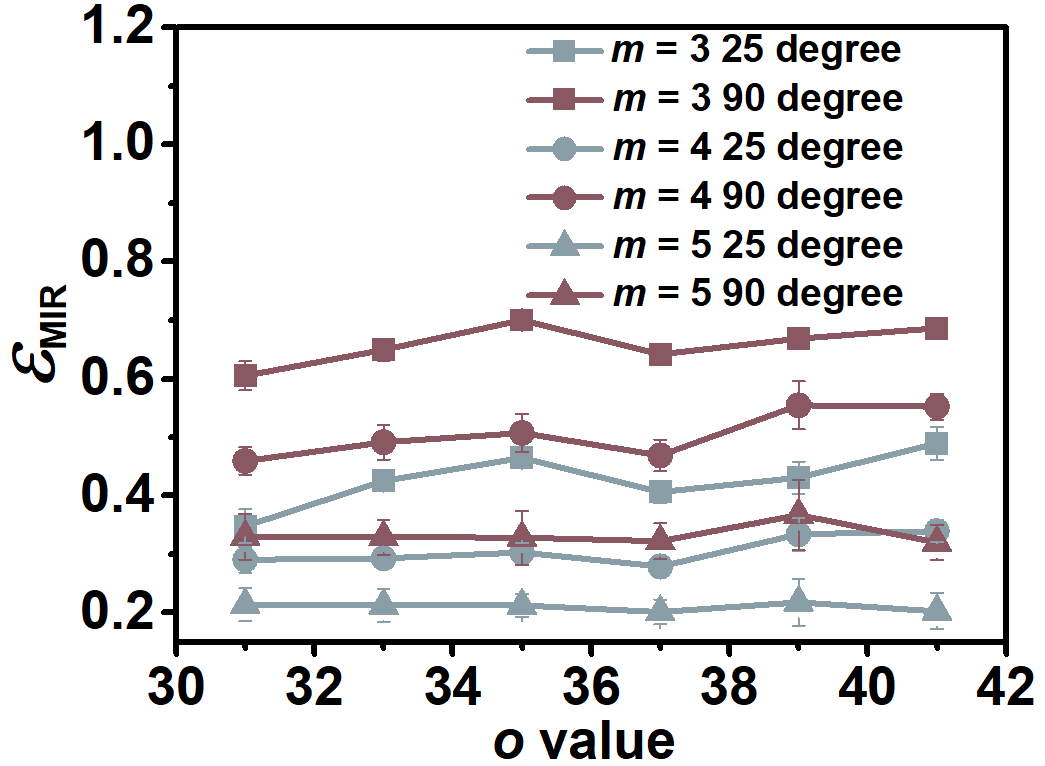


Figure S5. *ε*_MIR_ of the glass/(PEI/AgNWs)_m_/(PDDA/PAA)_o_/(PDDA/PAA/PDDA

/VO_2_)_5_ superstructures with varying *m* and *o* values at 25 ^o^C and 90 ^o^C.


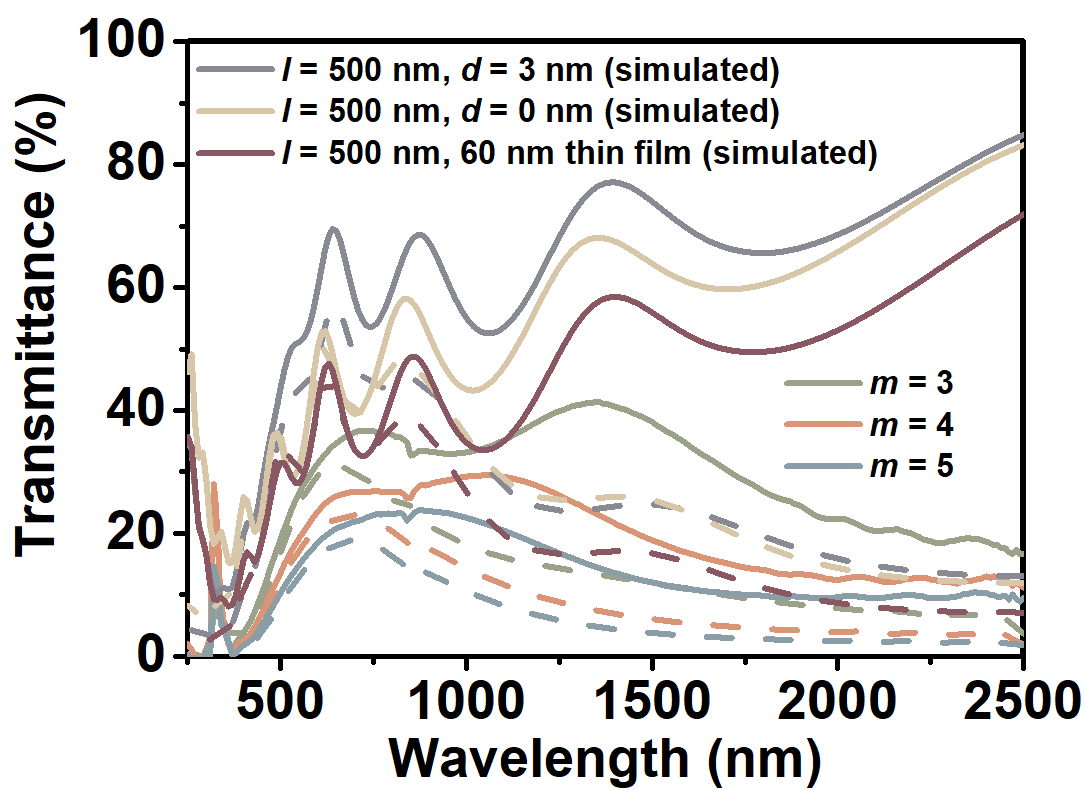


Figure S6. Comparison of simulated UV-vis-NIR transmittance spectra for the superstructure (*l* = 500 nm, *d* = 3 nm), the superstructure (*l* = 500 nm, *d* = 0 nm), and the superstructure (*l* = 500 nm, 60 nm VO_2_ thin film) and the measured transmittance spectra for the glass/(PEI/AgNWs)_m_/(PDDA/PAA)_39_/(PDDA/PAA

/PDDA/VO_2_)_5_ superstructures with varying *m* at 25 ^o^C (solid line) and 90 ^o^C (dashed line).

Table S1. *T*_lum_ and Δ*T*_sol_ for the superstructure (*l* = 500 nm, *d* = 3 nm), the superstructure (*l* = 500 nm, *d* = 0 nm), and the superstructure (*l* = 500 nm, 60 nm VO_2_ thin film).

| Properties | *T*_lum_ | Δ*T*_sol_ |
| --- | --- | --- |
| *l* = 500 nm, *d* = 3 nm | 48.6% | 19.2% |
| *l* = 500 nm, *d* = 3 nm | 37.1% | 11.4% |
| *l* = 500 nm, 60 nm thin film | 33.7% | 9.69% |


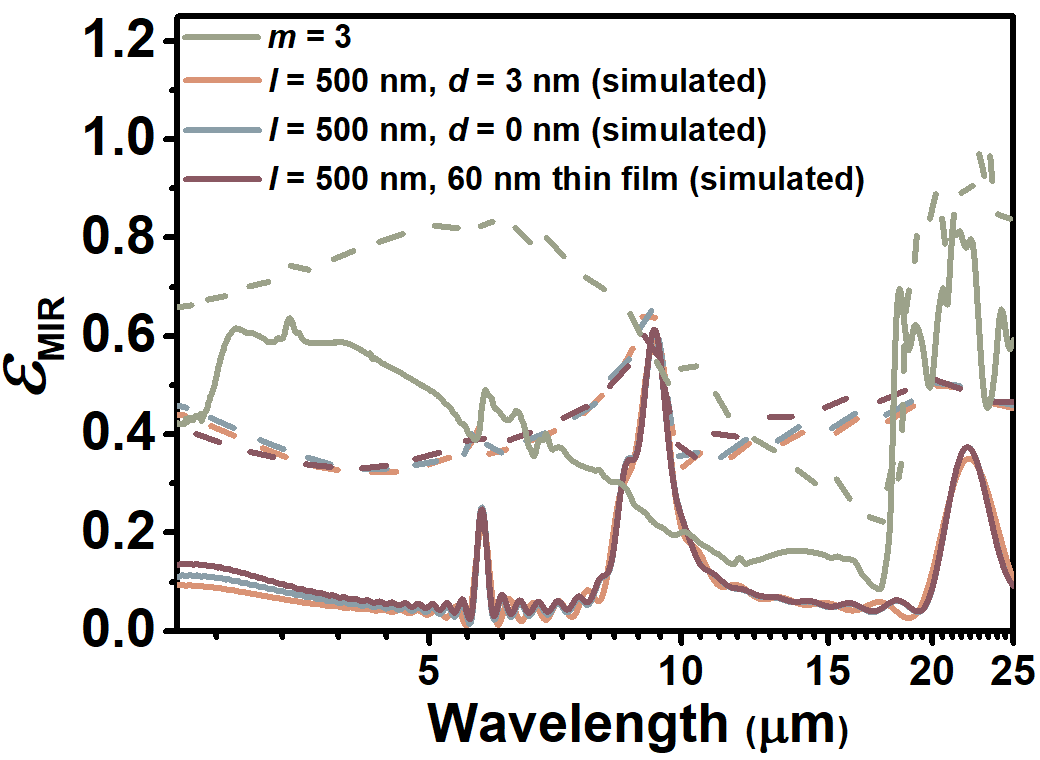


Figure S7. Comparison of *ε*_MIR_ for the superstructure (*l* = 500 nm, *d* = 3 nm), the superstructure (*l* = 500 nm, *d* = 0 nm), and the superstructure (*l* = 500 nm, 60 nm VO_2_ thin film) and the measured *ε*_MIR_ for the glass/(PEI/AgNWs)_3_/(PDDA

/PAA)_39_/(PDDA/PAA/PDDA/VO_2_)_5_ superstructure at 25 ^o^C (solid line) and 90 ^o^C (dashed line).


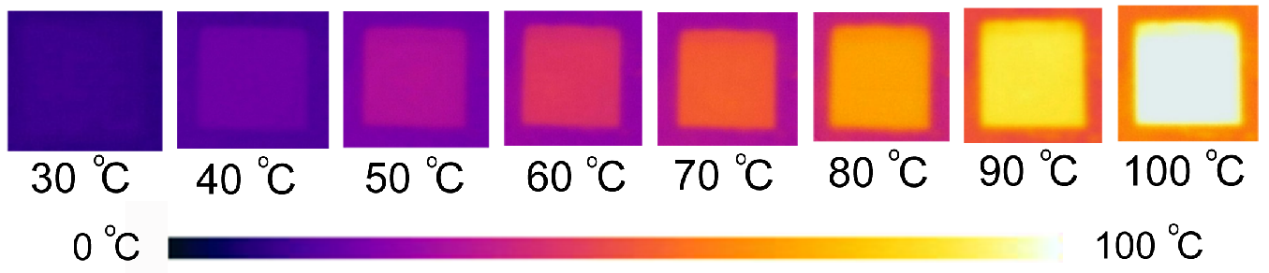


Figure S8. IR camera images of the glass/(PDDA/PAA)_39_/(PDDA/PAA/PDDA

/VO_2_)_5_ sample, captured at various temperatures, with a background *ε*_MIR_ of 0.5.


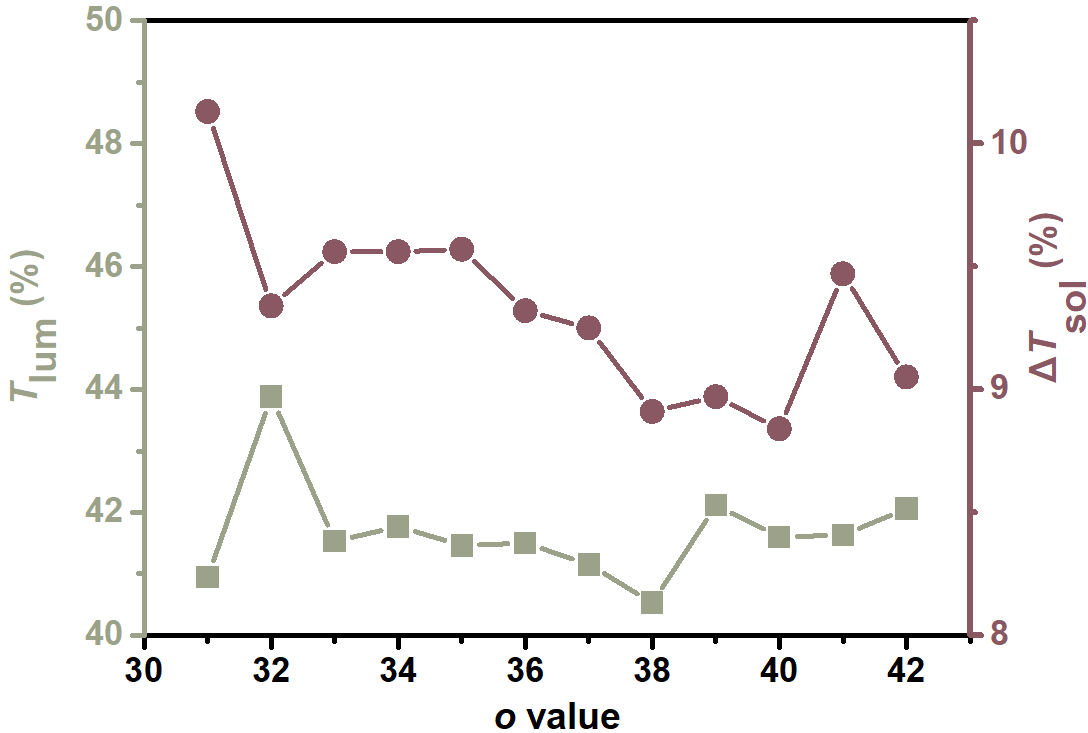


Figure S9. *T*_lum_ and Δ*T*_sol_ of the ITO/(PDDA/PAA)_o_/(PDDA/PAA/PDDA/VO_2_)_5_ superstructures with varying *o* (PDDA concentration: 0.25 mg/mL; PAA concentration: 2 mg/mL).


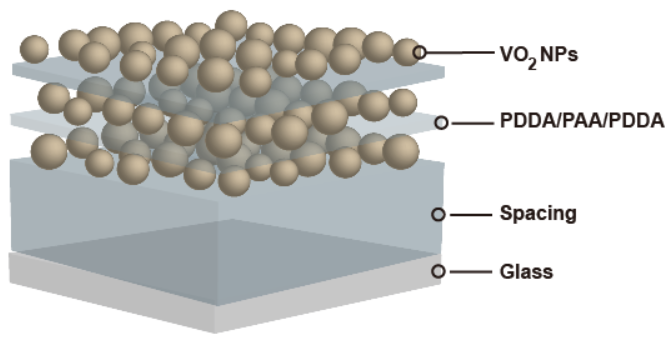


Figure S10. Schematic representation of the glass/(PDDA/PAA)_o_/(PDDA/PAA

/PDDA/VO_2_)_n_ superstructure.


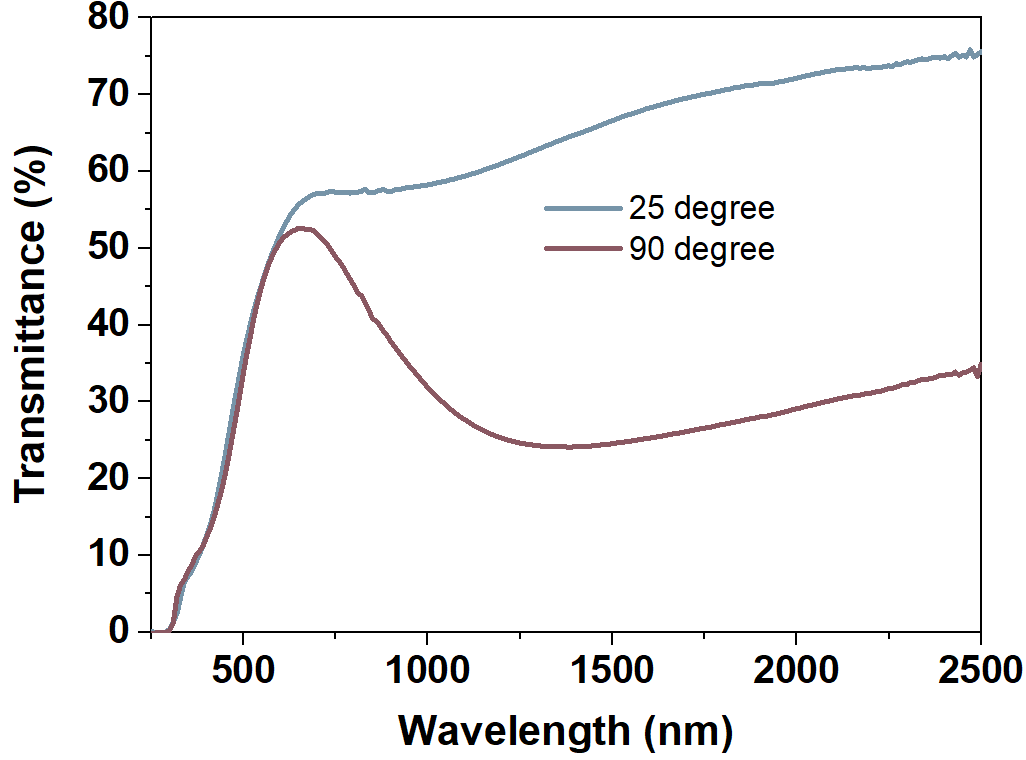


Figure S11. Transmittance spectra of the glass/(PDDA/PAA)_39_/(PDDA/PAA

/PDDA/VO_2_)_5_ superstructure at 25 and 90 ^o^C. (PDDA concentration: 0.25 mg/mL; PAA concentration: 2 mg/mL).


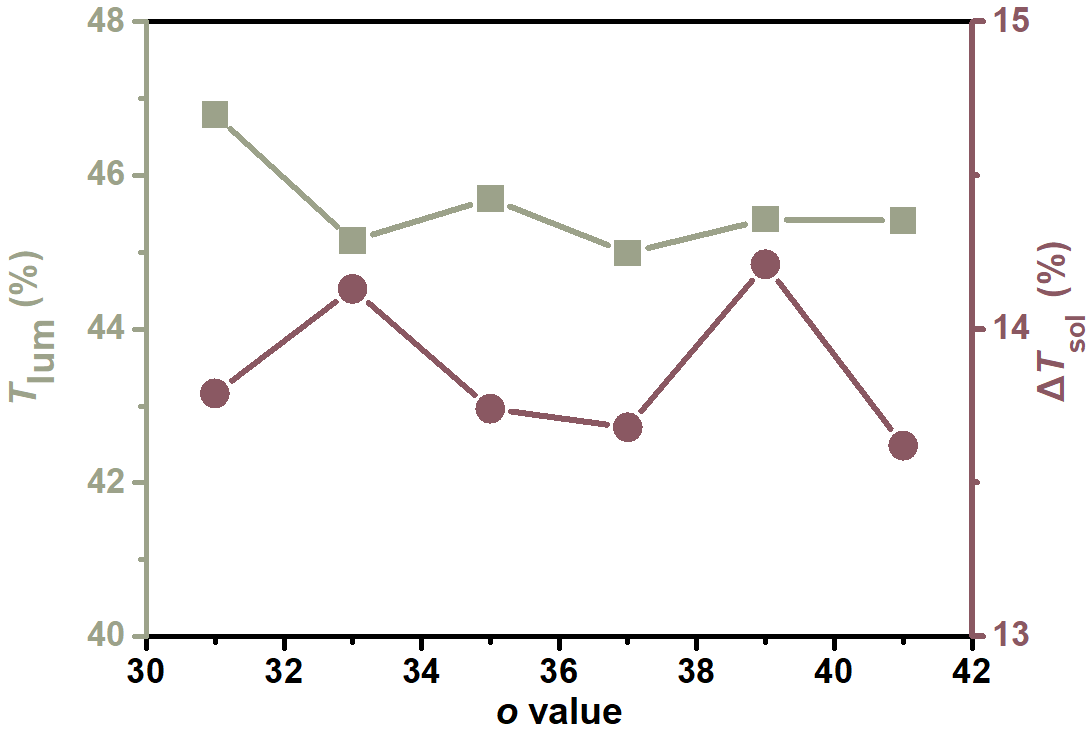


Figure S12. *T*_lum_ and Δ*T*_sol_ of the glass/(PDDA/PAA)_o_/(PDDA/PAA/PDDA

/VO_2_)_5_ superstructures with varying *o* (PDDA concentration: 0.25 mg/mL; PAA concentration: 2 mg/mL).


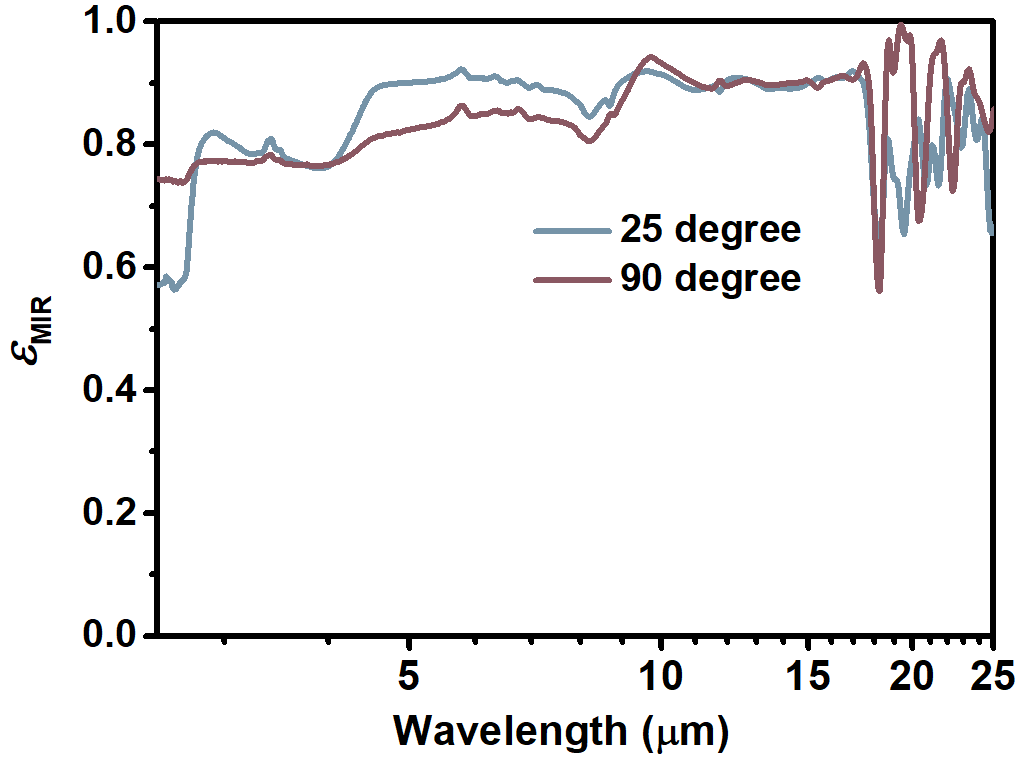


Figure S13. IR spectra of the glass/(PDDA/PAA)_39_/(PDDA/PAA/PDDA/VO_2_)_5_ superstructure at 25 ^o^C and 90 ^o^C (PDDA concentration: 0.25 mg/mL; PAA concentration: 2 mg/mL).

Table S2**.** Optical properties of normal glass, commercial low *ε*_MIR_ glass, and LbL-assembled RC window used in the energy-saving simulation.

| Properties | | Normal glass | Commercial low *ε*_MIR_ glass | LbL-assembled RC window |
| --- | --- | --- | --- | --- |
| Thickness (mm) | 3 | | 3 | 1 |
| *T*_lum_ | 88.9% | | 65.6% | 26.92% |
| *R*_lum, Front_ | 6.5% | | 7.6% | 13.49% |
| *R*_lum, Back_ | 6.5% | | 6.1% | 17.34% |
| *T*_sol_ | 83.6% | | 51.1% | 27.47% |
| *R*_sol, Front_ | 6.2% | | 8.3% | 17.18% |
| *R*_sol, Back_ | 6.2% | | 7.4% | 21.13% |
| ε_MIR, Front_ | 0.89 | | 0.4 | 0.431 |
| ε_MIR, Back_ | 0.89 | | 0.89 | 0.89 |
